# UV-B induces the expression of flavonoid biosynthetic pathways in blueberry (*Vaccinium corymbosum*) calli

**DOI:** 10.3389/fpls.2022.1079087

**Published:** 2022-11-22

**Authors:** Yan Song, Bin Ma, Qingxun Guo, Lianxia Zhou, Changyi Lv, Xiaoming Liu, Jianlei Wang, Xintong Zhou, Chunyu Zhang

**Affiliations:** Department of Horticulture, College of Plant Science, Jilin University, Changchun, China

**Keywords:** RNA sequencing, UV-B radiation, flavonoid, blueberry, transcription, plant hormone signal transduction

## Abstract

Ultraviolet-B (UV-B) radiation is an environmental signal that affects the accumulation of secondary metabolites in plants. In particular, UV-B promotes flavonoid biosynthesis, leading to improved fruit quality. To explore the underlying molecular mechanism, we exposed blueberry (*Vaccinium corymbosum*) calli to UV-B radiation and performed a transcriptome deep sequencing (RNA-seq) analysis to identify differentially expressed genes (DEGs). We detected 16,899 DEGs among different treatments, with the largest number seen after 24 h of UV-B exposure relative to controls. Functional annotation and enrichment analysis showed a significant enrichment for DEGs in pathways related to plant hormone signal transduction and phenylpropanoid and flavonoid biosynthesis. In agreement with the transcriptome data, flavonol, anthocyanin and proanthocyanidin accumulated upon UV-B radiation, and most DEGs mapping to the phenylpropanoid and flavonoid biosynthetic pathways using the KEGG mapper tool were upregulated under UV-B radiation. We also performed a weighted gene co-expression network analysis (WGCNA) to explore the relationship among genes involved in plant hormone signal transduction, encoding transcription factors or participating in flavonoid biosynthesis. The transcription factors VcMYBPA1, MYBPA2.1, MYB114, MYBA2, MYBF, and MYB102 are likely activators, whereas MYB20, VcMYB14, MYB44, and VcMYB4a are inhibitors of the flavonoid biosynthetic pathway, as evidenced by the direction of correlation between the expression of these MYBs and flavonoid biosynthesis-related genes. The transcription factors bHLH74 and bHLH25 might interact with MYB repressors or directly inhibited the expression of flavonoid biosynthetic genes to control flavonoid accumulation. We also observed the downregulation of several genes belonging to the auxin, gibberellin and brassinosteroid biosynthetic pathways, suggesting that MYB inhibitors or activators are directly or indirectly regulated to promote flavonoid biosynthesis under UV-B radiation.

## Introduction

Plants are constantly exposed to changing environmental conditions, among which ultraviolet-B (UV-B) radiation (280–315 nm) is an important factor that limits plant growth and development (Dotto and Casati, 2017). Plants accumulate flavonoids that protect against potential damage caused by UV exposure; importantly, flavonoids are also economically important compounds in fruits, as they possess nutritional benefits for human health (Norberto et al., 2013). Thus, despite the possible damage it can induce, the controlled application of UV-B radiation of fruits and other crops has been proposed as a means to improve fruit quality and their antioxidant contents (Henry-Kirk et al., 2018; González-Villagra et al., 2020).

Flavonoid compounds (flavonol, anthocyanin, and proanthocyanidin) are synthesized by the general phenylpropanoid biosynthetic pathway; the expression of the structural genes encoding the corresponding enzymes is upregulated by UV-B radiation and stimulates the accumulation of flavonoid compounds (Xie et al., 2011; Martínez-Lüscher et al., 2014). The UV-B-induced signaling pathway involves the UV-B photoreceptor UV resistance locus 8 (UVR8), the E3 ubiquitin ligase constitutively photomorphogenic 1 (COP1) and the basic zipper (bZIP) TF elongated hypocotyle (HY5) and zinc finger TF B-box domain containing protein (BBX) (Rizzini et al., 2011). These proteins direct or indirect regulate the transcription levels of regulators and flavonoid biosynthetic genes to affect flavonoid accumulation (Qiu et al., 2018; Bai et al., 2019). Among these regulators, four subgroups (4, 5, 6, and 7) from the 22 existing R2R3-MYB transcription factor gene family subgroups play regulatory roles in the biosynthesis of flavonoid compounds. More specifically, subgroup 4 encodes suppressors of flavonoid biosynthesis, while subgroups 5, 6, and 7 encode positive regulators of flavonoid biosynthesis (Kranz et al., 1998; Plunkett et al., 2018; Zhang et al., 2021). For example, the subgroup 4 *FaMYB1* from strawberry (*Fragaria ananassa*) suppressed anthocyanin and flavonol accumulation and *VvMYBC2-L1* from grapevine (*Vitis vinifera*) suppressed proanthocyanidin biosynthesis (Aharoni et al., 2001; Cavallini et al., 2015). At the same time bHLH transcription factor also effected flavonoid biosynthesis by interacting with MYB TFs or regulating structural genes of flavonoid biosynthesis (Matus et al., 2010; Zhu et al., 2020). UV-B also affects the expression of MYB transcription factor genes. For example, in Arabidopsis (*Arabidopsis thaliana*), the expression of *MYB111*, *MYB12*, and *production of anthocyanin pigment 1* (*PAP1*) is upregulated in response to UV-B radiation (Heijde and Ulm, 2018). In grapevine berries, UV-B triggers the upregulation of *flavonol synthase 1* (*VvFLS1*) *via* VvMYBF1 from subgroup 7, leading to a strong increase in flavonol concentration (Martínez-Lüscher et al., 2014). In apple (*Malus domestica*), the expression of *MdMYB10* from subgroup 6 and *MdMYB22* from subgroup 7 is downregulated throughout fruit development under reduced UV radiation and influences anthocyanin and flavonol production (Henry-Kirk et al., 2018). Thus, transcription factors have important roles in accumulating of UV-B-induced flavonoids.

Plant hormones also play critical parts in helping plants adapt to adverse environmental conditions (Yu et al., 2020). The growth-promoting hormones auxin, gibberellins, and brassinosteroids negatively regulate UV-B stress tolerance (Hectors et al., 2012; Roro et al., 2017; Liang et al., 2020) and inhibit flavonoid accumulation *via* direct interaction of some constituent proteins of their signalling pathway with MYBs or by binding to the promoters of *MYB* or structural genes involved in flavonoid biosynthesis to modulate their expression (Zhang Y. et al., 2017; Tan et al., 2019; Liang et al., 2020). However, it is unclear which genes related to plant hormone signalling participate in UV-B-induced flavonoid biosynthesis.

Blueberries (*Vaccinium corymbosum*) are often referred to as a “superfood” due to the health benefits associated with the phenylpropanoid compounds they contain, particularly anthocyanins, proanthocyanidins and flavonols (Ribera et al., 2010; Norberto et al., 2013). In recent years, several structural genes involved in flavonoid biosynthesis have been cloned and characterized in blueberry (Zhang et al., 2016; Zhang C. et al., 2017). Regulatory genes involved in this process have also been described, such as VcMYBA and VcMYBPA1, which regulate anthocyanin and proanthocyanidin biosynthesis, respectively (Zifkin et al., 2012; Plunkett et al., 2018). UV-B induces anthocyanin biosynthesis in the peel of harvested blueberry fruits by upregulating the expression of the structural and regulatory genes *VcMYB21* and *VcR2R3MYB*. UV-B radiation also increases flavonoid accumulation and promotes *VcMYBPA1* expression, but the underlying mechanism is unknown (Nguyen et al., 2017; González-Villagra et al., 2020).

In this study, we identified genes that are differentially expressed in blueberry calli in response to UV-B irradiation through transcriptome deep sequencing (RNA-seq) analysis and functional annotation of these differentially expressed genes (DEGs). To elucidate the mechanism driving flavonoid biosynthesis in blueberry fruits, we also assembled a regulatory network encompassing the plant hormone signal transduction pathways, transcription factor genes, and the flavonoid biosynthesis pathway by deploying weighted gene co-expression network analysis (WGCNA). The results presented here will broaden our understanding of UV-B-induced flavonoid biosynthesis and provide basic information on how to improve blueberry quality *via* simple exposure to UV-B light.

## Materials and methods

### Plant materials and stress treatments

White loose blueberry (*Vaccinium corymbosum*) calli were obtained from the cultivar ‘Northland’, which was cultured on a modified woody plant medium with Murashige and Skoog vitamins containing 3.0 mg/L 2,4-dichlorophenoxyacetic acid (2,4-D), 30 g/L sucrose, and 7 g/L agar, pH 5.4 ± 0.2. All calli were cultured under a 16-h light/8-h dark photoperiod at 25°C and subcultured every 21 days. UV-B was applied by means of narrow band lamps (TL20/01; 311-nm Philips, Netherlands) positioned above the calli at the height of about 10 cm. The calli were harvested right before (0 h) and after treatment consisting of 1, 3, 6, 12, and 24 h of UV-B radiation, frozen in liquid nitrogen and stored at –80°C. All samples were collected as three independent biological replicates and were labeled 0h_1, 0h_2, 0h_3, 1h_1, 1h_2, 0h_3, 3h_1, 3h_2, 3h_3, 6h_1, 6h_2, 6h_3, 12h_1, 12h_2, 12h_3, 24h_1, 24h_2, and 24h_3, respectively, for RNA-seq analysis. For reverse transcription quantitative PCR (RT-qPCR) and measurements of flavonol, anthocyanin, and proanthocyanidin contents (using the 0 h and 24 h samples), each biological replicate was assessed as three technical replicates.

### Transcriptome sequencing

Total RNAs were extracted with TRIzol reagent (Invitrogen, USA) and RNA concentration and purity was measured using a NanoDrop 2000 (Thermo Fisher Scientific, Wilmington, DE). RNA integrity was assessed using an RNA Nano 6000 Assay Kit for the Agilent Bioanalyzer 2100 system (Agilent Technologies, CA, USA). Sequencing libraries were generated using a NEBNext UltraTM RNA Library Prep Kit for Illumina (NEB, USA). The libraries were sequenced on an Illumina HiSeq 2500 platform, and 150 bp paired-end reads were generated. Raw reads in fastq format were processed through in-house perl scripts to obtain clean data. Q20, Q30, GC-content, and the sequence duplication level of the clean reads were calculated. The clean reads were mapped to the reference *Vaccinium corymbosum* cv. Draper V1.0 genome sequence (https://www.vaccinium.org/genomes) using Hisat2 software.

### Differential expression analysis and functional annotation

Gene expression levels were quantified as fragments per kilobase of transcript per million fragments mapped (FPKM) values. Differentially expressed genes (DEGs) resulting from the comparison of the 0 h sample to the 1, 3, 6, 12, and 24 h samples were identified using the criteria of absolute log_2_(fold change) ≥1 and a false discovery rate (FDR) < 0.01 by DEGSeq2. Gene function was annotated based on the following databases: Clusters of Orthologous Groups (COG) (Tatusov et al., 2000), Gene Ontology (GO) (Ashburner et al., 2000), Kyoto Encyclopedia of Genes and Genomes (KEGG) (Kanehisa et al., 2004), Eukaryotic Orthologous Groups (KOG) (Koonin et al., 2004), NCBI non-redundant protein sequences (NR) (Deng et al., 2006), Protein family (Pfam) (Finn et al., 2014), Swiss-Prot (A manually annotated and reviewed protein sequence database) (Apweiler et al., 2004) and evolutionary genealogy of genes: Non-supervised Orthologous Groups (eggNOG) (Huerta-Cepas et al., 2017). KEGG pathway enrichment analysis of DEGs was implemented with KOBAS software (Mao et al., 2005). The phenylpropanoid and flavonoid biosynthetic pathways were mapped against the phenylpropanoid, flavonoid, flavonol, and anthocyanin KEGG pathways using the DEGs identified above. Heatmap representations of gene expression levels were drawn using log_10_(FPKM) with the Tbtools (v1.098761) software (Chen et al., 2020).

### Weighted gene co-expression network analysis

WGCNA was performed with the WGCNA package in R (Langfelder and Horvath, 2008). First, the WGCNA algorithm assumes that the gene network follows a scale-free distribution, defines the gene co-expression correlation matrix and the adjacency function formed by the gene network, and then calculates the correlation coefficients of different nodes, based on which WGCNA builds a hierarchical clustering tree. The different branches of the clustering tree represent different gene modules. The co-expression degree of genes in significant individual modules is high, while the co-expression degree of genes belonging to different modules is low. Genes with different expression levels were assigned to various modules *via* a dynamic tree cut. There were at least 30 genes per co-expression module. Correlations among various modules were calculated using 0.25 as the similarity threshold. For genes in each module, KEGG pathway enrichment analysis was conducted to reveal the biological functions of each module. The genes within the WGCNA kMEblue module were selected to screen for enrichment of the plant hormone signal transduction pathway, transcription factor genes, and flavonoid biosynthetic pathway genes, and to draw the corresponding expression heatmap and regulatory network of the flavonoid pathway. Pearson’s correlation coefficients were compared using SPSS 19.0 software.

### RNA-Seq data validation

Total RNA was extracted from each sample using an RNA Extraction Kit (Sangon Biotech, Shanghai, China). RT-qPCR was performed on an ABI 7900HT real-time PCR system. Ten genes of interest (*4CL2*, *VcCHI3*, *DFR*, *VcF3’5’H*, *VcF3H-2*, *VcLAR*, *MYB44*, *MYB114*, *VcMYBPA1*, and *ARF18*) involved in flavonoid biosynthesis and belonging to the WGCNA kMEblue module were selected for analysis, using *GAPDH* (AY123769) as the reference transcript. Primer sequences are shown in **Table S1**. The relative expression levels of each gene were calculated using the 2^–ΔΔCt^ method.

### Measurement of flavonoid contents

The contents of flavonoid compounds including flavonols, anthocyanins, and proanthocyanidins for calli at 0 h and 24 h UV-B treatment were determined as described by Yang et al. (2022a). Each sample was ground to a powder and 0.5 g was extracted in 5 mL 80% (v/v) methanol at 4°C for 2 h to isolate the flavonols. The mixtures were centrifuged (8,000 g, 10 min, 4°C), 1 mL of the supernatant was removed and mixed with 1 mL methanol, and then 0.1 mL of 10% (w/v) aluminum chloride, 0.1 mL 1 M KOAc, and 2.8 mL water were added and the mixtures was incubated for 30 min at 25°C. Rutin was used as a master standard and the absorbance at 415 nm was measured. To measure total anthocyanin contents, samples (0.5 g) were extracted in 3 mL of 1% HCl in methanol and incubated at 4°C for 16 h. After centrifugation (8,000 g, 10 min, 4°C), a 2-mL supernatant was diluted with 2 mL water and the absorbance was measured at 530 nm and 650 nm. Total anthocyanin content was calculated using a previously published formula (Rabino and Mancinelli, 1986). Proanthocyanidin was detected using the DMACA method. Briefly, samples (0.5 g) were extracted in 5 mL of 70% (v/v) acetone containing 0.1% (w/v) ascorbic acid at 4°C for 30 min. After centrifugation (8,000 *g*, 10 min, 4°C), a 3-mL supernatant aliquot was extracted with 3 mL of ether at −20°C for 1 h. Then, 2 mL of the lower phase of the extracted liquid was removed and mixed with 1 mL of methanol and 0.5 mL 2% (w/v) DMACA solution. The mixture was incubated for 20 min at 25°C, and the absorbance was then measured at 643 nm. Catechin was used as the master standard (Wang et al., 2017).

## Results

### Transcriptome sequencing analysis

To reveal the regulatory network underlying the blueberry response to UV-B radiation, we performed an RNA-seq analysis using total RNA extracted from blueberry calli exposed to UV-B radiation for 1, 3, 6, 12, or 24 h. **Table 1** summarizes the details of all RNA-seq samples; we obtained 5.85–8.66 Gb of clean bases for each sample, with a Q30 ranging from 93.70 to 94.97% and a GC content ranging from 46.35 to 46.90%. We successfully mapped approximately 90.88 to 91.90% of all clean reads per sample to the blueberry reference genome.

**Table 1:** Summary of sequencing data.

Samples	Clean reads	Clean bases	GC Content	≥ Q30	Mapped reads
0h_1	2.78 × 10^7^	8.26 × 10^9^	46.4%	94.2%	91.1%
0h_2	2.70 × 10^7^	8.06 × 10^9^	46.6%	94.0%	91.2%
0h_3	2.23 × 10^7^	6.64 × 10^9^	46.5%	94.9%	91.3%
1h_1	2.76 × 10^7^	8.19 × 10^9^	46.8%	94.2%	90.9%
1h_2	1.99 × 10^7^	5.92 × 10^9^	46.8%	94.5%	90.9%
1h_3	2.60 × 10^7^	7.75 × 10^9^	46.9%	94.2%	91.0%
3h_1	2.60 × 10^7^	7.76 × 10^9^	46.9%	94.2%	91.1%
3h_2	2.65 × 10^7^	7.90 × 10^9^	46.9%	93.7%	91.1%
3h_3	2.69 × 10^7^	8.04 × 10^9^	46.8%	94.2%	91.3%
6h_1	2.90 × 10^7^	8,66 × 10^9^	46.6%	94.1%	91.6%
6h_2	2.43 × 10^7^	7.22 × 10^9^	46.9%	95.0%	91.7%
6h_3	2.15 × 10^7^	6.39 × 10^9^	46.8%	94.4%	91.5%
12h_1	2.48 × 10^7^	7.38 × 10^9^	46.9%	94.5%	91.7%
12h_2	2.09 × 10^7^	6.21 × 10^9^	46.9%	94.7%	91.3%
12h_3	1.96 × 10^7^	5.85 × 10^9^	46.5%	94.6%	91.6%
24h_1	2.52 × 10^7^	7.52 × 10^9^	46.65%	94.6%	91.9%
24h_2	2.06 × 10^7^	6.10 × 10^9^	46.7%	94.8%	91.8%
24h_3	2.59 × 10^7^	7.71 × 10^9^	46.8%	94.5%	91.9%

### Differential expression gene analysis

**Figure 1:**
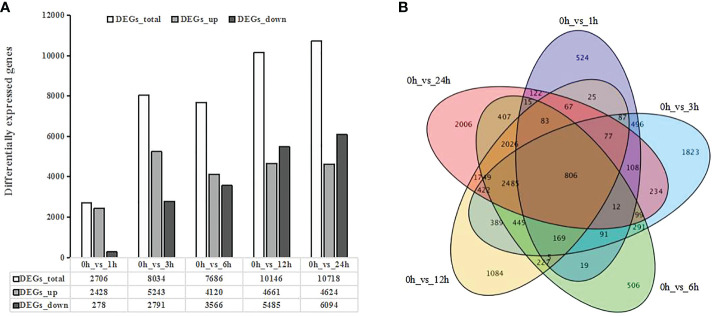
Differentially expressed genes (DEGs) in response to UV-B treatment in blueberry revealed by transcriptome deep sequencing (RNA-seq). **(A)** Number of DEGs in response to UV-B. DEGs_up, upregulated DEGs; DEGs_down, downregulated DEGs. **(B)** Venn diagrams showing the extent of overlaps between DEGs across pairwise comparisons. Purple, DEGs for 0 h vs 1 h; blue, DEGs for 0 h vs 3 h; green, DEGs for 0 h vs 6 h; yellow, DEGs for 0 h vs 12 h; and pink, DEGs for 0 h vs 24 h Q-value < 0.01.

To investigate changes in gene expression under UV-B radiation, we identified differentially expressed genes (DEGs) between the control samples (0 h) and each time point of the UV-B treatment, which returned a total of 16,899 DEGs (**Table S2**). We detected 2,706 DEGs in 0h_vs_1h, of which 2,428 were upregulated and only 278 were downregulated. The number of DEGs increased gradually with longer UV-B exposure of up to 6 h, with downregulated DEGs remaining scarcer than upregulated DEGs. The number of DEGs further increased to 10,146 in 0h_vs_12h and to 10,718 in 0h_vs_24h, respectively, but now with more downregulated DEGs than upregulated DEGs (**Figure 1A**). A Venn diagram comparing all the lists of DEGs shows that 806 DEGs are shared by all pairwise comparisons (**Figure 1B**). These results indicate that the number of DEGs increases with the duration of UV-B radiation treatment.

### Functional annotation and enrichment analysis of DEGs

We annotated the function of DEGs in response to UV-B radiation with the COG, GO, KEGG, KOG, NR, Pfam, Swiss-Prot, and eggNOG databases (**Table S3**). During UV-B treatment, the number of annotated DEGs increased, starting at 2,596 annotated DEGs out of 2,706 after 1 h, and rising to 9,953 annotated DEGs out of 10,718 after 24 h. The number of annotated DEGs also differed by database, with the highest number obtained with the NR database and the lowest with the COG database.

We then undertook a functional classification of DEGs that were annotated in the COG, eggNOG, and KOG databases (**Table S4**). We observed that DEGs are most enriched in terms related to signal transduction mechanisms, secondary metabolite biosynthesis, transport and catabolism, as well as transcription, especially in the eggNOG and KOG functional annotations. Furthermore, we performed a GO classification of shared annotated DEGs for the three categories biological process, cellular component, and molecular function (**Figure S1**; **Table S5**). In the biological process category, cellular processes and metabolic processes were the most enriched; in the cellular components category, cell and cell part were the most enriched; and in the molecular functions category, binding and catalytic activity were the most enriched.

**Figure 2:**
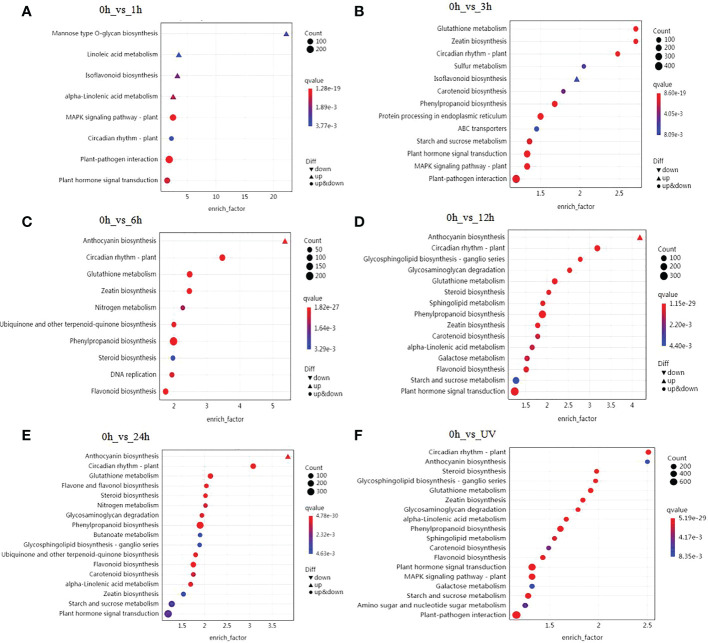
Summary of KEGG pathway enrichment analysis. Analysis of significantly enriched DEGs after 1 h **(A)**, 3 h **(B)**, 6 h **(C)**, 12 h **(D)**, or 24 h **(E)** of UV-B radiation compared to 0 h The number of DEGs is indicated by the size of the circle, and the solid upper triangle and inverted triangle represent upregulated and downregulated DEGs, respectively. **(F)** Analysis of significantly enriched DEGs comparing the control samples (0 h) to all samples exposed to UV-B.

To further elucidate the biological functions of the DEGs, we carried out a KEGG pathway enrichment analysis. We identified 136 KEGG pathways whose encoded proteins are affected during UV-B treatment in blueberry calli (**Table S6**). Using a *Q*-value < 0.01 as cutoff, we determined that the KEGG pathway circadian rhythm-plant pathway was significantly affected at all time points of UV-B treatment (**Figure 2**). Similarly, the plant hormone signal transduction pathway was significantly enriched at almost all stages, suggesting that plant hormones are involved in the response to UV-B stress. We also established that many DEGs are involved in the biosynthesis of phenylpropanoid-derived compounds. Among them, genes participating in the isoflavonoid biosynthetic pathway were induced after 1 and 3 h UV-B treatment (**Figures 2A, B**). Likewise, genes from the phenylpropanoid biosynthetic pathway began to be enriched after 3 h of UV-B treatment (**Figures 2B–E**), followed by genes from the anthocyanin and flavonoid biosynthetic pathways, starting at 6 h of UV-B treatment (**Figures 2C–E**). We conclude that genes involved in the biosynthesis of phenylpropanoid-derived compounds exhibit a precise spatial pattern: the expression of isoflavonoid biosynthetic genes is induced first, followed by genes involved in the phenylpropanoid pathway, and finally genes involved in the anthocyanin and flavonoid biosynthetic pathways. In agreement with this result, the top 20 enriched pathways identified when comparing the control samples (0 h) to any sample exposed to UV-B included anthocyanin biosynthesis, phenylpropanoid biosynthesis, and flavonoid biosynthesis (**Figure 2F**).

### UV-B radiation induces flavonoid accumulation

**Figure 3:**
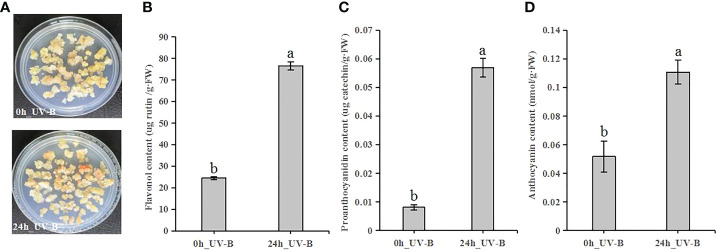
UV-B exposure induces flavonoid accumulation in blueberry calli. **(A)** Representative blueberry calli before (0 h) and after (24 h). **(B-D)** Contents of flavonol **(B)**, proanthocyanidin **(C)** and anthocyanin **(D)** after UV-B radiation for 0 h or 24 h Error bars indicate ± SD of the mean of three independent biological replicates. Different letters indicate significant differences (*p* < 0.05) among samples by Tukey’s test.

To elucidate the effects of UV-B radiation on the biosynthesis of flavonoid compounds, we measured the flavonoid contents of blueberry calli treated by UV-B radiation for 24 h (**Figure 3A**). The contents of flavonols, proanthocyanidins, and anthocyanins increased 3.1-, 7.0-, and 2.1-fold, respectively, after 24 h of UV-B irradiation relative to the control samples (**Figures 3B–D**). Thus, UV-B radiation promotes the accumulation of flavonoid compounds.

### The phenylpropanoid and flavonoid biosynthetic KEGG pathways under UV-B radiation

We screened for genes involved in the phenylpropanoid, flavonoid, flavonol, and anthocyanin KEGG pathways among the DEGs. We identified 73 genes encoding 32 types of enzymes, based on Enzyme Commission (EC) annotated data (**Table 2**). We then plotted the expression levels of these genes as a heatmap. Again, the genes selected here clustered as a function of the duration of UV-B exposure, with the 0, 1, and 3 h treatments forming one group and the 6, 12, and 24 h treatments forming another (**Figure S2**). Notably, the later induction of these DEGs suggested that the accumulation of phenylpropanoid metabolites mainly occurs after a longer UV-B exposure.

**Table 2:** The regulation of DEGs involved in flavonoid biosynthesis under UV-B radiation.

Enzymes	Gene orhomologue name	Gene ID	0h_vs_1h		0h_vs_3h		0h_vs_6h		0h_vs_12		0h_vs_24h	
			log_2_(FC)^a^	regulated^b^	log_2_(FC)	regulated	log_2_(FC)	regulated	log_2_(FC)	regulated	log_2_(FC)	regulated
PAL [EC:4.3.1.24]	*PAL1*	VaccDscaff24-augustus-gene-101.15	-0.69	normal	-0.13	normal	2.04	up	2.28	up	2.87	up
	*PAL3*	VaccDscaff10-augustus-gene-222.29	-0.49	normal	0.13	normal	1.76	up	1.75	up	1.82	up
C4H [EC:1.14.14.91]	*C4H*	VaccDscaff11-augustus-gene-343.38	0.57	normal	1.16	up	1.03	up	1.34	up	1.31	up
4CL [EC:6.2.1.12]	*4CL2*	VaccDscaff34-processed-gene-57.9	-0.47	normal	0.03	normal	1.72	up	1.72	up	2.06	up
	*4CL2-like*	VaccDscaff28-augustus-gene-349.38	–^c^	–	–	–	–	–	0.58	normal	1.28	up
	*4CL6*	VaccDscaff47-augustus-gene-2.17	-0.23	normal	-1.68	down	-1.79	down	-2.25	down	-2.82	down
HCT [EC:2.3.1.133]	*HST*	VaccDscaff17-processed-gene-329.4	1.02	up	1.00	up	0.20	normal	-0.10	normal	0.22	normal
	*SHT*	VaccDscaff38-augustus-gene-0.16	-0.15	normal	1.00	normal	1.89	up	0.99	normal	0.85	normal
C3’H [EC:1.14.14.96]	*C3’H*	VaccDscaff10-snap-gene-65.38	–	–	–	–	1.22	up	1.18	up	1.55	up
F5H [EC:1.14.-.-]	*F5H*	VaccDscaff123-augustus-gene-2.26	0.62	normal	1.80	up	1.09	normal	1.09	normal	0.85	normal
COMT [EC:2.1.1.68]	*COMT-like*	VaccDscaff19-augustus-gene-150.25	-0.44	normal	-0.63	normal	-0.88	normal	-1.81	down	-2.47	down
	*COMT1*	VaccDscaff40-augustus-gene-215.28	-0.42	normal	-0.55	normal	-0.39	normal	-1.56	down	-1.51	down
CCoAOMT [EC:2.1.1.104]	*CCoAOMT*	VaccDscaff45-snap-gene-216.26	0.27	normal	0.58	normal	-0.19	normal	-0.86	normal	-1.08	down
CCR [EC:1.2.1.44]	*VcCCR*	VaccDscaff4-processed-gene-398.7	-0.04	normal	-0.18	normal	0.82	normal	0.24	normal	1.13	up
	*CCR1-like*	VaccDscaff6-augustus-gene-409.27	-0.88	normal	-1.09	normal	0.50	normal	0.82	normal	1.52	up
	*CCR*	VaccDscaff12-processed-gene-15.8	0.44	normal	1.58	up	1.12	up	1.12	up	0.79	normal
	*CCR2-like*	VaccDscaff7-processed-gene-8.8	-0.17	normal	-0.84	normal	-0.06	normal	-0.86	normal	-1.04	down
	*CCR2*	VaccDscaff23-snap-gene-364.44	0.62	normal	2.06	up	1.63	up	1.82	up	1.43	up
	*CCR-like*	VaccDscaff2-augustus-gene-63.19	-0.41	normal	-0.88	normal	1.12	up	0.88	normal	1.93	up
	*CCR1*	VaccDscaff3-processed-gene-55.9	-0.16	normal	-0.77	normal	-0.71	normal	-1.04	down	-1.03	down
CAD [EC:1.1.1.195]	*CAD1*	VaccDscaff14-snap-gene-183.27	0.53	normal	2.52	up	2.72	up	2.73	up	2.38	up
	*CAD6*	VaccDscaff12-snap-gene-1.33	-0.54	normal	-1.52	down	-0.97	normal	-1.66	down	-1.66	down
	*CAD9*	VaccDscaff13-augustus-gene-6.23	-0.72	normal	0.28	normal	0.62	normal	0.67	normal	1.03	up
CHS [EC:2.3.1.74]	*CHS*	VaccDscaff42-augustus-gene-14.30	-0.90	normal	1.40	up	5.28	up	5.35	up	6.33	up
	*CHS1*	VaccDscaff3-augustus-gene-334.17	-0.70	normal	-0.74	normal	0.98	normal	1.04	up	1.14	up
CHI [EC:5.5.1.6]	*CHI*	VaccDscaff21-processed-gene-199.1	-0.44	normal	-0.29	normal	1.31	up	1.02	normal	-0.02	normal
	*CHI3*	VaccDscaff21-augustus-gene-108.29	-0.42	normal	-0.68	normal	1.63	up	2.25	up	2.57	up
DFR [EC:1.1.1.219 1.1.1.234]	*VcDFR*	VaccDscaff13-processed-gene-166.8	-0.79	normal	-0.64	normal	0.70	normal	1.00	up	1.27	up
	*DFR*	VaccDscaff1613-processed-gene-0.0	0.09	normal	1.02	up	1.19	up	0.61	normal	0.56	normal
F3H [EC:1.14.11.9]	*VcF3H2*	VaccDscaff16-augustus-gene-381.32	-0.71	normal	-0.63	normal	1.80	up	2.03	up	2.74	up
	*VcF3H1*	VaccDscaff13-augustus-gene-41.36	-0.48	normal	-0.67	normal	0.64	normal	1.16	up	1.33	up
F3’5’H [EC:1.14.14.81]	*F3’5’H2*	VaccDscaff29-augustus-gene-305.28	-0.93	normal	0.03	normal	1.09	up	1.83	up	1.50	up
	*VcF3’5’H*	VaccDscaff10-augustus-gene-348.25	0.07	normal	-0.96	normal	1.13	up	0.72	normal	2.67	up
F3’H [EC:1.14.14.82]	*F3’H1*	VaccDscaff32-augustus-gene-159.26	-0.46	normal	-0.88	normal	1.24	up	1.57	up	2.03	up
	*F3’H*	VaccDscaff43-snap-gene-241.31	0.09	normal	-0.41	normal	-1.02	down	-0.98	normal	-0.96	normal
FLS [EC:1.14.20.6]	*FLS1*	VaccDscaff6-augustus-gene-163.26	-0.49	normal	-0.74	normal	-0.25	normal	0.70	normal	1.06	up
	*VcFLS*	VaccDscaff25-augustus-gene-225.24	–	–	1.29	normal	2.99	up	4.08	up	4.53	up
LDOX [EC:1.14.20.4]	*VcLDOX*	VaccDscaff43-augustus-gene-236.29	-0.41	normal	-0.79	normal	0.24	normal	0.63	normal	1.04	up
	*LDOX-like*	VaccDscaff33-processed-gene-213.10	0.31	normal	1.52	up	0.46	normal	–	–	-0.50	normal
UFGT [EC:2.4.1.115]	*VcUFGT*	VaccDscaff20-augustus-gene-68.33	1.10	up	4.39	up	6.85	up	7.33	up	7.86	up
UGT75 [EC:2.4.1.298]	*UGT75C1*	VaccDscaff4-processed-gene-124.9	0.44	normal	1.30	up	1.26	up	1.11	up	1.43	up
LAR [EC:1.17.1.3]	*LAR-like*	VaccDscaff35-augustus-gene-247.32	-0.56	normal	-1.52	down	-0.96	normal	-1.73	down	-1.09	down
	*VcLAR*	VaccDscaff2-augustus-gene-298.20	-0.94	normal	0.55	normal	1.69	up	1.82	up	2.13	up
ANR [EC:1.3.1.77]	*ANR*	VaccDscaff15-augustus-gene-178.21	-0.55	normal	-1.26	down	-0.28	normal	0.20	normal	0.32	normal
	*ANR-like*	VaccDscaff44-augustus-gene-19.33	0.42	normal	1.24	up	1.09	up	0.81	normal	0.62	normal
F6H [EC:1.14.11.61]	*F6H1-2*	VaccDscaff21-augustus-gene-269.37	-0.07	normal	1.18	up	0.52	normal	0.88	normal	0.47	normal
FGGT1 [EC:2.4.1.239 2.4.1.-]	*GT1*	VaccDscaff38-processed-gene-219.4	–	–	–	–	3.86	up	2.81	up	3.28	up
POD [EC:1.11.1.7]	*PERP7*	VaccDscaff8-processed-gene-129.6	0.87	normal	1.35	up	-0.01	normal	-1.09	down	-2.42	down
	*PER15*	VaccDscaff44-augustus-gene-228.32	-0.13	normal	-0.74	normal	-1.87	down	-1.36	down	0.66	normal
	*PERP7-like*	VaccDscaff88-snap-gene-4.29	0.52	normal	1.32	up	-0.55	normal	-0.29	normal	-2.12	down
PS [EC:2.4.1.357]	*UGT88A1-like*	VaccDscaff21-snap-gene-49.64	0.17	normal	-1.82	down	-1.28	down	-1.52	down	-1.05	down
	*UGT88B1-like*	VaccDscaff21-processed-gene-49.13	-0.85	normal	-1.22	down	-0.41	normal	-0.55	normal	-0.80	normal
SGT [EC:2.4.1.128]	*SGT*	VaccDscaff14-processed-gene-356.18	0.38	normal	2.80	up	2.87	up	3.10	up	1.27	up
UGT29 [EC:2.4.1.236]	*UGT29-like1*	VaccDscaff1-processed-gene-272.1	-0.62	normal	-0.80	normal	-1.10	down	-1.92	down	-2.48	down
	*UGT29*	VaccDscaff29-augustus-gene-198.28	0.51	normal	2.28	up	0.95	normal	1.67	up	1.17	up
	*UGT94-like2*	VaccDscaff21-processed-gene-210.10	–	–	3.22	up	3.22	up	3.77	up	4.40	up
	*UGT94-like3*	VaccDscaff28-snap-gene-281.34	-0.10	normal	1.29	up	1.67	up	1.25	up	1.41	up
	*UGT94-like4*	VaccDscaff28-augustus-gene-280.39	0.39	normal	1.29	up	1.09	up	0.99	normal	1.19	up
UGT73 [EC:2.4.1.-]	*UGT73C6*	VaccDscaff33-processed-gene-116.6	0.25	normal	0.60	normal	1.21	up	1.27	up	1.78	up
ALDH2 [EC:1.2.1.68]	*ALDH2C4*	VaccDscaff69-augustus-gene-3.25	0.32	normal	0.61	normal	-0.53	normal	-0.69	normal	-2.49	down
BGLU [EC:3.2.1.21]	*GH3BG3*	VaccDscaff10-snap-gene-111.37	-0.41	normal	-0.33	normal	-0.49	normal	-1.07	down	-1.07	down
	*GH3B*	VaccDscaff21-snap-gene-299.24	0.49	normal	0.80	normal	0.40	normal	-0.66	normal	-1.00	down
	*GH3BG1*	VaccDscaff33-augustus-gene-101.21	0.26	normal	0.79	normal	-0.53	normal	-1.49	down	-1.93	down
	*GH3BG5*	VaccDscaff15-augustus-gene-315.29	0.57	normal	1.61	up	1.49	up	1.33	up	1.04	up
	*BGLU41*	VaccDscaff1-snap-gene-432.37	1.14	up	0.60	normal	0.77	normal	0.87	normal	0.72	normal
	*BGLU11-like*	VaccDscaff46-snap-gene-198.38	0.07	normal	-0.60	normal	-0.17	normal	-0.97	normal	-1.17	down
	*BGLU40-like*	VaccDscaff35-processed-gene-230.7	0.13	normal	-0.83	normal	-1.09	down	-2.11	down	-1.21	down
	*BGLU42*	VaccDscaff17-snap-gene-224.18	-0.21	normal	-0.78	normal	-0.90	normal	-1.07	down	-0.87	normal
	*BGLU42-like*	VaccDscaff27-augustus-gene-205.16	-0.19	normal	-0.56	normal	-0.67	normal	-1.27	down	-0.67	normal
	*BGLU44*	VaccDscaff27-processed-gene-4.10	0.50	normal	0.61	normal	0.57	normal	0.69	normal	1.80	up
	*GH3BG5*	VaccDscaff221-processed-gene-1.6	0.55	normal	-0.51	normal	-0.48	normal	-0.28	normal	-1.24	down
CSE [EC:3.1.1.-]	*CSE*	VaccDscaff42-augustus-gene-210.23	0.76	normal	1.01	up	0.11	normal	0.44	normal	0.25	normal
	*CSE-like*	VaccDscaff27-augustus-gene-309.30	0.51	normal	0.49	normal	1.05	up	0.89	normal	1.15	up

a log_2_(fold change).

b upregulation or downregulation.

c No expression.

**Figure 4:**
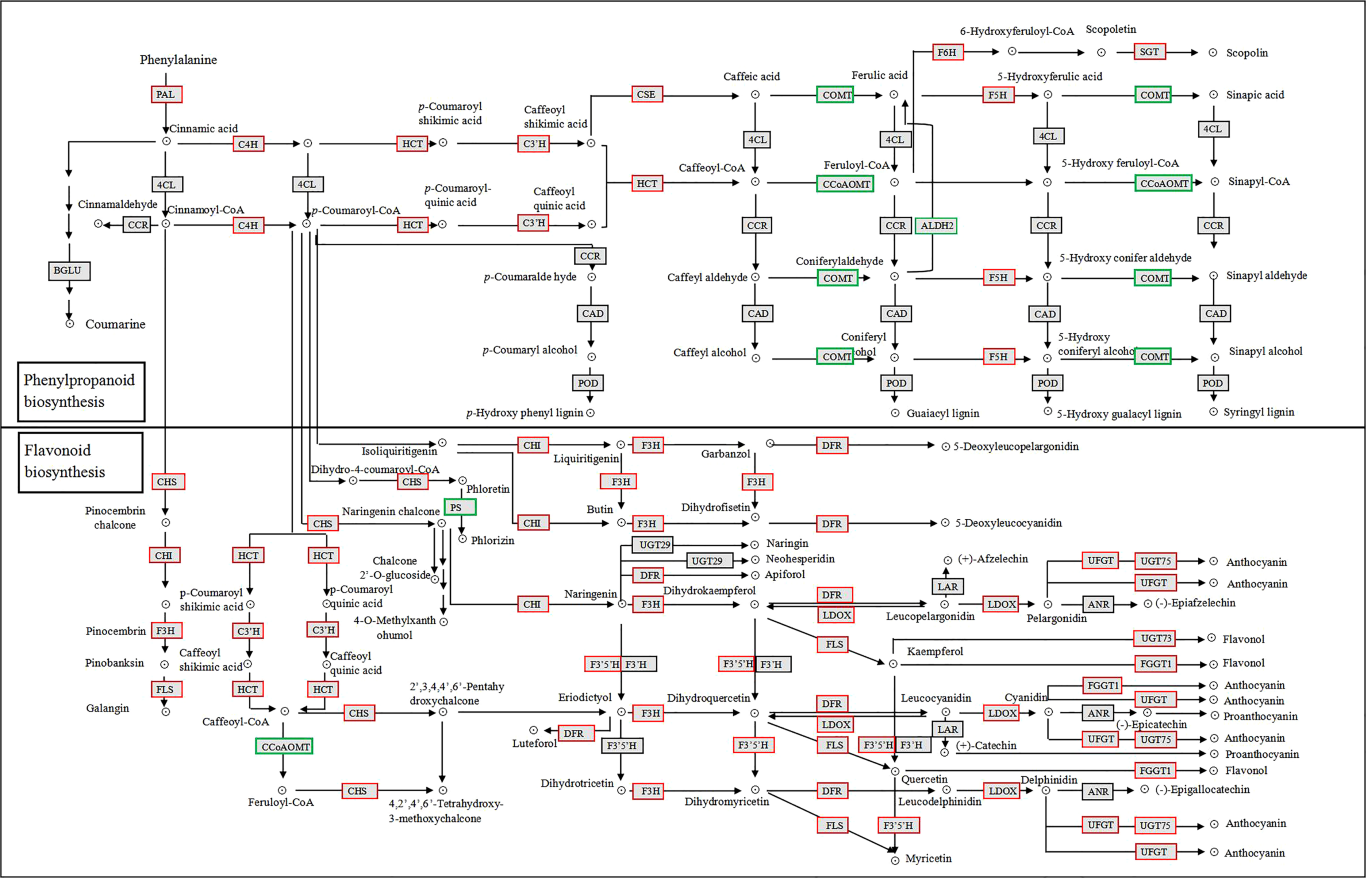
Phenylpropanoid and flavonoid KEGG biosynthetic pathways in blueberry calli under UV-B radiation. Red and green boxes represent upregulated and downregulated DEGs, respectively.

We mapped the 73 DEGs identified above onto the KEGG phenylpropanoid and flavonoid biosynthetic pathways (**Figure 4**). Among them, only three genes were induced after 1 h of UV-B radiation, and many genes were induced after 3 h. Most of the genes, including *phenylalanine ammonia lyase* (*PAL1*), *PAL3*, *4-coumarate CoA ligase* (*4CL2*), *chalcone synthase synthase* (*CHS*), *chalcone isomerase* (*CHI3*), *VcFLS*, and *VcUFGT*, were induced under UV-B radiation, and their expression reached the highest levels at 24 h (**Table 2**). Most genes involved in the phenylpropanoid biosynthetic pathway were rapidly upregulated, especially *PAL1* and *4CL2* from 6 h of UV-B radiation treatment onwards. By contrast, *4CL6* was rapidly downregulated from 3 h of UV-B radiation onwards. Most genes involved in the flavonoid biosynthetic pathway were upregulated; *CHS* expression was induced from 3 h of UV-B radiation treatment onwards, reaching a log_2_(FC) value of 6.33 at 24 h. The expression of *F3’5’H*, *F3’H*, and *FLS*, which are involved in the flavonol biosynthetic pathway, was upregulated under UV-B radiation. Among genes involved in the anthocyanin biosynthetic pathway, *VcUFGT* expression was induced from 1 h of UV-B exposure onwards, and then rapidly upregulated from 3 h onwards, reaching the highest level at 24 h (7.86 for log_2_(FC) value). *VcLAR*, which is involved in the proanthocyanin biosynthesis pathway, was also upregulated from 6 h onwards, reaching a log_2_(FC) value of 2.13 at 24 h (**Table 2**). These results indicate that the expression of most genes involved in phenylpropanoid metabolite biosynthesis is induced under UV-B radiation and that *PAl1*, *4CL2*, *CHS*, *VcFLS*, *VcUFGT*, and *VcLAR* play important roles in UV-B-induced flavonoid accumulation.

### Weighted gene co-expression network analysis identifies gene modules associated with flavonoid metabolism under UV radiation

**Figure 5:**
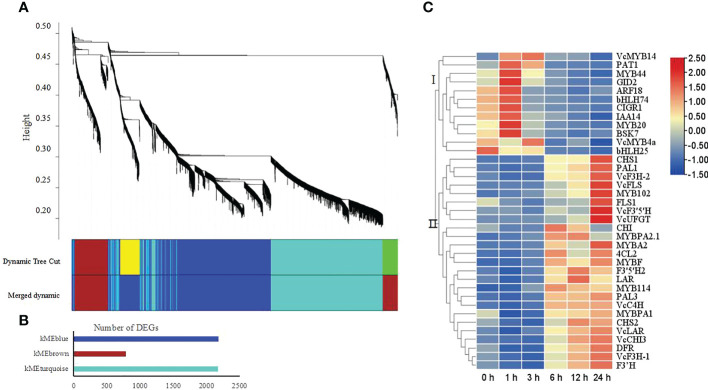
Weighted gene co-expression network analysis (WGCNA) of DEGs obtained from all pairwise comparisons. **(A)** Hierarchical clustering tree showing the co-expression modules identified by WGCNA. Different modules are marked with different colors. Each leaf of the cluster tree represents a gene. **(B)** Number of assigned DEGs to the different modules. **(C)** Heatmap representation of hierarchical clustering analysis of identified genes involved in the phenylpropanoid, flavonoid, and anthocyanin metabolic pathways, in the plant hormone signal transduction pathway, and encoding transcription factors in the kMEblue module. Blue, low expression; red, high expression, based on log_2_(FPKM).

To reveal the regulatory network underlying flavonoid metabolism under UV radiation, we conducted a weighted gene co-expression network analysis (WGCNA) using the DEGs defined above. WGCNA clustered the DEGs into three modules, namely, kMEblue, kMEbrown, and kMEturquoise, which contained 2,174, 783, and 2,171 genes, respectively (**Figures 5A, B**). KEGG enrichment analysis showed that the kMEblue module comprises many of the genes involved in the phenylpropanoid, flavonoid, and anthocyanin metabolism pathways (**Figure S3**). We thus characterized the connection between these genes and those associated with plant hormone signal transduction, or encoding transcription factors by looking for their annotations in the KEGG, Swiss-Prot, and NR databases (**Table S7**). We identified 37 genes fulfilling the above criteria, and their expression levels fell into two groups, as evidenced by a heatmap (**Figure 5C**). Genes from group I were downregulated, whereas genes from group II were upregulated under UV-B radiation; the latter group included all flavonoid metabolism genes. We also identified ten MYB transcription factor genes in the kMEblue module; these genes belonged to subgroups 4 (*VcMYB4a*), 11 (*MYB102*), 8 (*MYB20*), 5 (*MYBA2.1* and *VcMYBPA*), 2 (*VcMYB14*), 7 (*MYBF*), 6 (*MYBA2* and *VcMYB144*), and 22 (*MYB44*) (**Figure S4**).

To investigate the functions of these MYB transcription factor genes in the flavonoid pathway, we calculated the Pearson’s correlation coefficients (*r*) between their expression levels and those of genes involved in flavonoid biosynthesis during UV-B radiation (**Table 3**). We observed that the expression of *MYBA2*, *MYBF*, *MYB102*, *MYBPA1*, *MYBPA2*.*1*, and *MYB114* is positively correlated with that of most genes involved in the flavonoid pathway. Conversely, the expression levels of *MYB20*, *VcMYB14*, *MYB44*, and *VcMYB4a* were negatively correlated with those of most flavonoid metabolism genes. We also observed a positive correlation between the expression levels of *bHLH74*, *VcMYB20*, and *VcMYB44* and between *bHLH25* and *VcMYB4a*, and a negative correlation between the expression levels of *bHLH74* and *MYB114*, and between the expression levels of *bHLH25* and those of *MYB114*, *MYBA2*, and *MYBF*. The expression of *bHLH74* and *bHLH25* was also negatively correlated with the expression levels of genes involved in flavonoid metabolism.

**Table 3:** Pearson’s correlation coefficients (*r*) between the transcription factor genes and DEGs from the phenylpropanoid metabolite pathway DEGs, plant hormone signal transduction pathway in the kMEblue module.

Genes	*PAL1*	*PAL3*	*VcC4H*	*4CL2*	*CHS1*	*CHS2*	*CHI*	*VcCHI3*	*VcF3H-1*	*VcF3H-2*	*F3’H*	*VcF3’5’H*	*F3’5’H*	*FLS1*	*VcFLS*	*DFR*	*VcUFGT*	*VcLAR*	*LAR*	*bHLH74*	*bHLH25*	*IAA14*	*ARF18*	*GID2*	*CIGR1*	*PAT1*	*BSK7*
*MYBPA1*	0.88*	0.88*	0.89*	0.80	0.83*	0.91*	0.61	0.88*	0.89*	0.92**	0.92*	0.69	0.86*	0.73	0.79	0.96**	0.69	0.92*	0.83*	-0.65	-0.62	-0.76	-0.64	-0.87*	-0.68	-0.99**	-0.75
*MYBPA2.1*	0.65	0.83*	0.87*	0.71	0.59	0.71	0.91*	0.70	0.63	0.66	0.68	0.22	0.89*	0.08	0.47	0.67	0.26	0.75	0.92**	-0.70	-0.80	-0.68	-0.74	-0.67	-0.70	-0.70	-0.74
*MYB114*	0.83*	0.95**	0.96**	0.90*	0.80	0.75	0.78	0.81	0.82*	0.77	0.20	0.51	0.92**	0.33	0.69	0.80	0.53	0.88*	0.89*	-0.84*	-0.91*	-0.84*	-0.84*	-0.78	-0.85*	-0.76	-0.85*
*MYBA2*	0.96**	0.92**	0.88*	0.97**	0.98**	0.75	0.41	0.86*	0.97**	0.87*	0.89*	0.91*	0.77	0.77	0.92*	0.89*	0.88*	0.90*	0.68	-0.70	-0.85*	-0.79	-0.63	-0.70	-0.72	-0.73	-0.68
*MYBF*	0.95**	0.99**	0.97**	0.98**	0.95**	0.81*	0.62	0.90*	0.95**	0.89*	0.91*	0.76	0.90*	0.61	0.86*	0.91*	0.76	0.95**	0.84*	-0.79	-0.92**	-0.85*	-0.75	-0.77	-0.80	-0.80	-0.79
*MYB102*	0.97**	0.87*	0.82*	0.84*	0.98**	0.81	0.19	0.91*	0.96**	0.91*	0.92*	0.94**	0.77	0.84*	0.99*	0.90*	0.97**	0.91*	0.68	-0.69	-0.82*	-0.81*	-0.61	-0.68	-0.70	-0.66	-0.65
*MYB20*	-0.78	-0.83*	-0.82*	-0.75	-0.74	-0.73	-0.56	-0.75	-0.76	-0.75	-0.75	-0.51	-0.84*	-0.47	-0.68	-0.79	-0.57	-0.86*	-0.79	0.96**	0.64	0.97**	0.96**	0.99**	0.97**	0.84*	0.99**
*VcMYB14*	-0.72	-0.69	-0.70	-0.63	-0.68	-0.81	-0.48	-0.73	-0.75	-0.81*	-0.80	-0.63	-0.67	-0.76	-0.66	-0.85*	-0.60	-0.74	-0.64	0.37	0.37	0.52	0.36	0.71	0.41	0.92**	0.52
*MYB44*	-0.86*	-0.89*	-0.89*	-0.80	-0.82*	-0.85*	-0.58	-0.85	-0.85*	-0.86*	-0.86*	-0.61	-0.89*	-0.60	-0.77	-0.90*	-0.66	-0.93*	-0.86*	0.88*	0.66	0.94**	0.88*	0.99**	0.90*	0.94**	0.94**
*VcMYB4a*	-0.88*	-0.89*	-0.88*	-0.89*	-0.89*	-0.78	-0.54	-0.85	-0.89*	-0.85*	-0.87*	-0.77	-0.78	-0.64	-0.82*	-0.85*	-0.73	-0.83*	-0.73	0.45	0.87*	0.56	0.40	0.48	0.47	0.67	0.46
*bHLH74*	-0.74	-0.80	-0.79	-0.73	-0.71	-0.64	-0.49	-0.70	-0.71	-0.66	-0.67	-0.46	-0.80	-0.34	-0.66	-0.70	-0.55	-0.81*	-0.76	—	—	—	—	—	—	—	—
*bHLH25*	-0.86*	-0.91*	-0.90*	-0.86*	-0.86*	-0.73	-0.54	-0.84*	-0.85*	-0.79	-0.81*	-0.64	-0.85*	-0.42	-0.80	-0.77	-0.67	-0.85*	-0.81	—	—	—	—	—	—	—	—
*IAA14*	—	—	—	—	—	—	—	—	—	—	—	—	—	—	—	—	—	—	—	—	—	—	0.95*	—	—	—	—
*GID2*	—	—	—	—	—	—	—	—	—	—	—	—	—	—	—	—	—	—	—	—	—	—	—	—	0.91*	0.91*	—

*Correlation significant at the 0.05 level. **Correlation significant at the 0.01 level. — Correlation analysis was not conducted.

**Figure 6:**
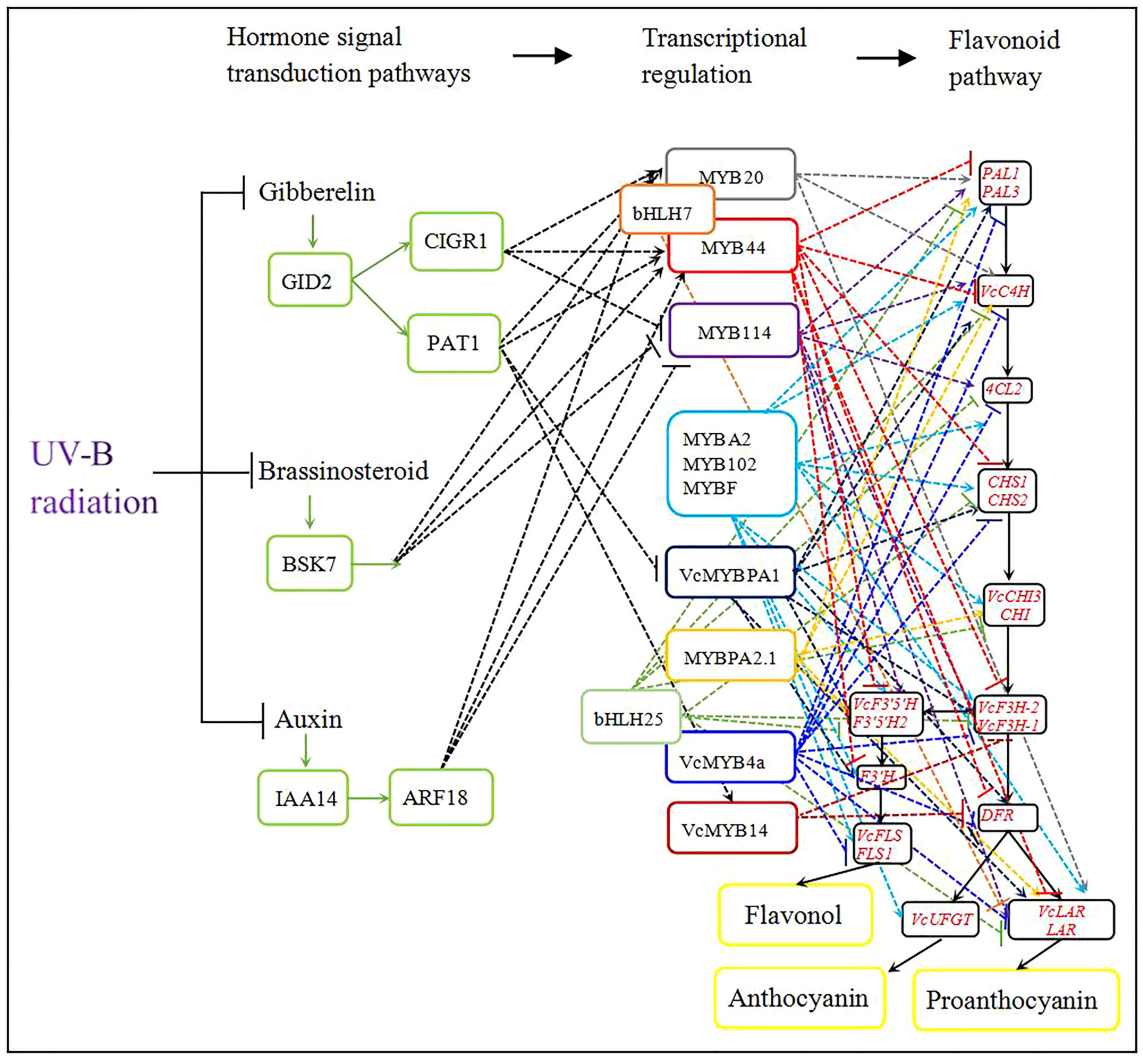
Model of the integration of UV-B-induced flavonoid accumulation with plant hormone signal transduction pathways. Black solid lines represent the flavonoid metabolic pathway and colored dashed lines represent regulatory pathways emanating from each transcription factor gene. In particular, green solid lines represent plant hormone signal transduction pathways and black dashed lines indicate MYB transcription factor genes that are regulated by a phytohormone. Arrows indicate activation; short lines indicate inhibition.

To elucidate the roles of plant hormones in regulating the flavonoid biosynthesis pathway, we calculated the correlation between *MYB* expression levels and the expression of plant-hormone-related genes (**Table 3**). In the auxin biosynthetic pathway, the expression levels of *indole-3-acetic acid inducible 14* (*IAA14*) and *auxin-response factor 18* (*ARF18*) were negatively correlated with those of *MYB114* and positively correlated with those of *MYB20* and *MYB44*. In the gibberellic acid biosynthetic pathway, c*hitin-inducible gibberellin-responsive protein 1* (*CIGR1*) expression was positively correlated with that of *MYB20* and *MYB44* and negatively correlated with that of *MYB114*. Scarecrow-like transcription factor (*PAT1*) expression was positively correlated with that of *MYB20*, *MYB44*, and *VcMYB14* and negatively correlated with that of *VcMYBPA1*. The expression level of *Ga insensitive dwarf 2* (*GID2*) was positively correlated with those of *CIGR1* and *PAT1*. However, among genes involved in the brassinosteroid biosynthetic pathway, only *brassinosteroid-signaling kinase KINASE 7* (*BSK7*) expression levels appeared to be positively correlated with *MYB20* and *MYB44* expression and negatively correlated with *MYB114* expression (**Table 3**). **Figure 6** summarizes the regulatory network of UV-B-induced flavonoid biosynthetic pathway through hormone signal transduction and transcriptional regulation pathways based on DEGs identified from the WGCNA kMEblue module in this study.

To validate the accuracy and reliability of the RNA-seq data, we selected ten genes of interest involved in flavonoid biosynthesis from the WGCNA kMEblue module for RT-qPCR analysis (**Figure S5**). A linear regression analysis showed a positive correlation between the RNA-seq and RT-qPCR results, with correlation coefficients (*R* ^2^) of 0.8926 (0 h vs 1 h), 0.8338 (0 h vs 3 h), 0.8849 (0 h vs 6 h), 0.8856 (0 h vs 12 h), and 0.8336 (0 h vs 24 h). This observation confirmed that the RNA-seq data in this study are accurate and reliable.

## Discussion

UV-B radiation is a major abiotic stress that triggers a variety of plant responses with consequences for plant growth, development, and accumulation of secondary metabolites that involve the actions of many genes (Dotto and Casati, 2017; Gupta et al., 2018; González-Villagra et al., 2020; Ding et al., 2021). For example, transcriptome profiling and DEG analysis have shown that UV-B radiation influences secondary metabolite biosynthesis, plant–pathogen interaction, and plant hormone signal transduction pathways in lettuce (*Lactuca sativa*) (Zhang et al., 2019) and plant hormone signal transduction pathways in horseweed (*Conyza lini*) (Zhan et al., 2021). In our study, functional annotation showed that genes from plant hormone signal transduction pathways and secondary metabolism biosynthetic pathways (including phenylpropanoid, flavonoid, and anthocyanin) were the most enriched among DEGs (**Table S4**; **Figure 2**). The timing of DEG appearance is important, as DEGs involved in plant hormone signal transduction pathways were significantly enriched after 1 h of UV-B exposure, followed by genes involved in the phenylpropanoid biosynthetic pathway after 3 h, and by genes involved in the flavonoid and anthocyanin biosynthetic pathways after 6 h of UV-B treatment. These results indicated that phytohormone-related genes may constitute the first response to UV-B radiation.

UV-B radiation affects the phenylpropanoid biosynthetic pathway and influences the accumulation of flavonoid compounds, especially flavonols, proanthocyanidins, and anthocyanins (Berli et al., 2011; Henry-Kirk et al., 2018). In this study, we showed that the flavonol, proanthocyanidin, and anthocyanin contents of blueberry calli significantly increased after 24 h of UV-B radiation, indicating that UV-B radiation promotes the accumulation of flavonoid compounds (**Figure 3**). In grapevine berries, the expression of *FLS1* and *UFGT* is induced by UV-B, leading to increased flavonol and anthocyanin concentration (González-Villagra et al., 2020). In lettuce, transcriptome analysis showed that the transcriptional upregulation of *C4H*, *4CL*, *CHS*, *FLS*, and *DFR* promoted anthocyanin biosynthesis in response to UV-B radiation (Zhang et al., 2019). Our study demonstrated that the expression of most genes involved in flavonoid biosynthesis was upregulated, especially *PAl1*, *4CL2*, *CHS*, *VcFLS*, *VcUFGT*, and *VcLAR* genes (**Figure 4**; **Table 2**). These genes are involved in the biosynthesis of flavonol, proanthocyanidin, or anthocyanin and may be the key genes for UV-B-induced flavonoid biosynthesis.

WGCNA has been widely used to identify gene regulatory networks from different KEGG pathways (Zhu et al., 2019; Li et al., 2022). The biosynthesis of flavonoid compounds is co-regulated by multiple genes (e.g., *PAL*, *4CL*, *CHI*, and *FLS*) during half-high blueberry fruit development (Yang et al., 2022b). In the current study, we assembled the regulatory network of the phenylpropanoid biosynthesis pathway under UV radiation using WGCNA. The transcriptome analysis showed that 19 genes encoding flavonoid biosynthetic enzymes are co-regulated to promote the accumulation of flavonols, proanthocyanidins, and anthocyanins (**Figure 6**).

R2R3-MYB transcription factors control the transcriptional regulation of flavonoid structural genes in various horticultural plants including blueberry (Zifkin et al., 2012; Plunkett et al., 2018). In this study, we systematically compared the expression levels of ten MYB transcription factor genes against those of structural genes from the flavonoid biosynthetic pathway. Among the encoded transcription factors, VcMYBPA1 and MYBPA2.1 (subgroup 5), MYB114 and MYBA2 (subgroup 6), and MYBF (subgroup 7) promoted the expression of genes involved in flavonoid biosynthesis. Subgroups 5, 6, and 7 positively regulate anthocyanin, proanthocyanidin, or flavonol biosynthesis (Bogs et al., 2007; Czemmel et al., 2009; Zifkin et al., 2012; An et al., 2015; Plunkett et al., 2018; Karppinen et al., 2021; Zhang et al., 2021). Thus, these MYBs may activate UV-B-induced flavonoid biosynthesis. MYB102 belongs to subgroup 11, which contributes to plant resistance against stress (Denekamp and Smeekens, 2003; Xu et al., 2015). The co-expression analysis showed that *VcMYB102* expression was positively correlated with that of genes involved in flavonoid biosynthesis. VcMYBPA1, MYBPA2.1, MYB114, MYBA2, MYBF, and MYB102 may activate the UV-B-induced flavonoid pathway.

VcMYB4a belongs to subgroup 4 and inhibits lignin biosynthesis (Yang et al., 2022a). In this study, *VcMYB4a* expression was also negatively correlated with that of genes from the flavonoid biosynthetic pathway and *VcMYB4a* likely encodes an inhibitor. Subgroups 22, 8, and 2 play important roles in plant responses to abiotic stresses. For example, AtMYB73 from subgroup 22 negatively regulates responses to salt stress, AtMYB20 from subgroup 8 negatively regulates plant response to drought stress; and AtMYB15 from subgroup 2 decreases tolerance to freezing stress (Agarwal et al., 2006; Kim et al., 2013; Gao et al., 2014). Thus, some MYB transcription factors from these subgroups function as negative regulators of abiotic stresses. Transcriptome analysis showed that the expression of *MYB44* (subgroup 22), *MYB20* (subgroup 8), and *VcMYB14* (subgroup 2) was negatively correlated with the expression of genes involved in flavonoid biosynthesis under UV-B radiation in blueberry calli (**Table 3**). Furthermore, MYB44, MYB20, and VcMYB14 inhibit the flavonoid pathway in response to UV-B radiation.

Basic helix-loop-helix (bHLH) family members also regulate flavonoid biosynthesis (Matus et al., 2010; Zhu et al., 2020). bHLH proteins directly regulate the expression of structural genes or interact with MYB proteins to regulate flavonoid accumulation (Hartmann et al., 2005; An et al., 2012). In Arabidopsis, bHLH74 regulates root growth and bHLH25 influences cyst nematode parasitism (Jin et al., 2011; Bao et al., 2014). In this study, the expression of *bHLHL74* homologs was positively correlated with that of *MYB20* and *MYB44*, while the expression of a *bHLH25* homolog was also positively correlated with that of *VcMYB4a* in blueberry calli under UV-B radiation. Importantly, *bHLHL74* and *bHLH25* expression levels were negatively correlated with those of genes involved in flavonoid biosynthesis (**Figure 6**; **Table 3**). Thus, the bHLHL74 and bHLH25 transcription factors may negatively regulate flavonoid biosynthesis by repressing the expression of genes involved in the flavonoid biosynthetic pathway or by interacting with MYB proteins under UV-B irradiation.

Plant hormones, including auxin, gibberellins, and brassinosteroids, play important roles in flavonoid biosynthesis (Jeong et al., 2004; Loreti et al., 2008; Peng et al., 2011). In Arabidopsis, gibberellins negatively regulate low temperature- or sucrose-induced anthocyanin accumulation and inhibit flavonol biosynthesis (Loreti et al., 2008; Zhang et al., 2011; Tan et al., 2019). The F-box protein, GID2 (also named SLEEPY1) positively regulates gibberellin signaling by promoting the polyubiquitination of DELLA proteins, subsequently leading to their degradation by the 26S proteasome. (Sasaki et al., 2003; McGinnis et al., 2003). DELLA, PAT1, and CIGR1 all belong to the GRAS family of transcriptional regulators. PAT1 is involved in phytochrome A signal transduction in Arabidopsis, while *OsCIGR1* expression is induced by exogenous gibberellins in rice (*Oryza sativa*) (Bolle et al., 2000; Day et al., 2004). DELLA proteins are repressors of gibberellin signaling. In the absence of GA, DELLA proteins accumulate and interact with MYB proteins to promote flavonol biosynthesis. In the presence of GA, the GA-dependent complex GA-GID1-DELLA promotes DELLA degradation *via* the 26S proteasome and then reduces the transcriptional activity of MYBs to inhibit flavonol biosynthesis (Tan et al., 2019). DELLA proteins also positively regulate nitrogen-deficiency-induced anthocyanin accumulation by directly interacting with PAP1 (Zhang Y. et al., 2017). Our study showed that the expression of *GID2*, *PAT1*, and *CIGR1* is downregulated under UV-B radiation; moreover, correlation analysis showed that *GID2* expression is positively correlated with those of *PAT1* and *CIGR1*, which are themselves positively correlated with the expression of *MYB20* and *MYB44* (**Figure 6**; **Table 3**). UV-B results in a decrease in endogenous gibberellin concentration (Roro et al., 2017), such that in the absence of gibberellin under UV-B exposure, the GA-GID2-PAT1 and GA-GID2-CIGR1 complexes are less abundant and can no longer repress MYB114 and VcMYBPA1 function, leading to the induction of *MYB20*, *MYB44*, and *VcMYB14* expression, and thus to flavonoid accumulation.

The phytohormone auxin inhibits anthocyanin biosynthesis and controls UV-mediated accumulation of flavonoids (Hectors et al., 2012; Ji et al., 2015). In apple, auxin regulates anthocyanin biosynthesis through the Aux/IAA-ARF signaling pathway. MdARF13 interacts with MdIAA121 and directly binds to the promoter of *MdDRF* to inhibit anthocyanin biosynthesis. MdARF13 also interacts with MdMYB10 to repress anthocyanin accumulation by downregulating *MdMYB10* expression (Wang et al., 2018). In Arabidopsis, ARF17 directly binds to the *MYB108* promoter to regulate anther dehiscence (Xu et al., 2019). In this study, *ARF18* and *IAA14* expression were repressed by UV-B radiation; we observed that *ARF18* expression was positively correlated with that of *IAA14*, *MYB20*, and *MYB44* and negatively correlated with that of *MYB114* under UV-B irradiation (**Figure 6**; **Table 3**). Thus, it is possible that UV-B radiation reduces endogenous auxin concentrations and diminishes the expression levels of *ARF18* and *IAA*, resulting in higher *MYB114* expression or lower *MYB20* and *MYB44* expression to induce flavonoid biosynthesis.

Brassinosteroids negatively affect plant tolerance of UV-B stress. BRI1-EMS-Suppressor 1 (BES1) acts downstream of brassinosteroid-insensitive 1 (BRI1) to promote UV-B-induced flavonol biosynthesis by binding to the promoters of *MYB11*, *MYB12*, and *MYB111* (Liang et al., 2020). BSK is a critical family of receptor-like cytoplasmic kinases acting in BR signal transduction, of which BSK3, BSK4, BSK7, and BSK8 belong to the same clade and functionally overlap (Tang et al., 2008; Sreeramulu et al., 2013). AtBSK3 interacts with BRI1, the phosphatase BRI1 suppressor 1 (BSU1), and the kinase brassinosteroid-insensitive 2 (BIN2) to regulate the BR signaling pathway; BSK8 interacts with the Kelch-type phosphatase BSL2 to regulate the activity of sucrose-phosphate synthase enzyme (Wu et al., 2014; Ren et al., 2019). However, the function of BSKs in the regulation UV-B-induced flavonoid biosynthesis is unclear. We used the WGCNA method to determine the role of *BSK7*. *BSK7* expression was downregulated and showed a significant association with the expression levels of genes encoding MYB proteins involved in flavonoid biosynthesis under UV-B radiation (**Figure 6**; **Table 3**). Therefore, BSK7 may negatively regulate UV-B-induced flavonoid biosynthesis; however, it is unclear whether BSKs regulate flavonoid biosynthesis by regulating MYB transcription factors or other pathways.

## Data availability statement

The original contributions presented in the study are publicly available. This data can be found here: NCBI, PRJNA892908.

## Author contributions

CZ conceived and designed the project. YS, BM, QG, LZ, CL, XL, JW and XZ participated in the experiments and analyzed the data. YS and BM drafted the manuscript. CZ modified the manuscript. All authors read and approved the final manuscript. All authors contributed to the article and approved the submitted version.

## Funding

This study was supported by the National Natural Science Foundation of China (grant number 31700260).

## Conflict of interest

The authors declare that the research was conducted in the absence of any commercial or financial relationships that could be construed as a potential conflict of interest.

## Publisher’s note

All claims expressed in this article are solely those of the authors and do not necessarily represent those of their affiliated organizations, or those of the publisher, the editors and the reviewers. Any product that may be evaluated in this article, or claim that may be made by its manufacturer, is not guaranteed or endorsed by the publisher.
