# Supplementary material for: UV-B induces the expression of flavonoid biosynthetic pathways in blueberry (Vaccinium corymbosum) calli
Source: Front Plant Sci. 2022 Nov 22;13:1079087. doi: 10.3389/fpls.2022.1079087 (PMC9722975; doi:10.3389/fpls.2022.1079087)
Supplement: Supplementary file 1 [file DataSheet_1.pdf]

0h_vs_1h

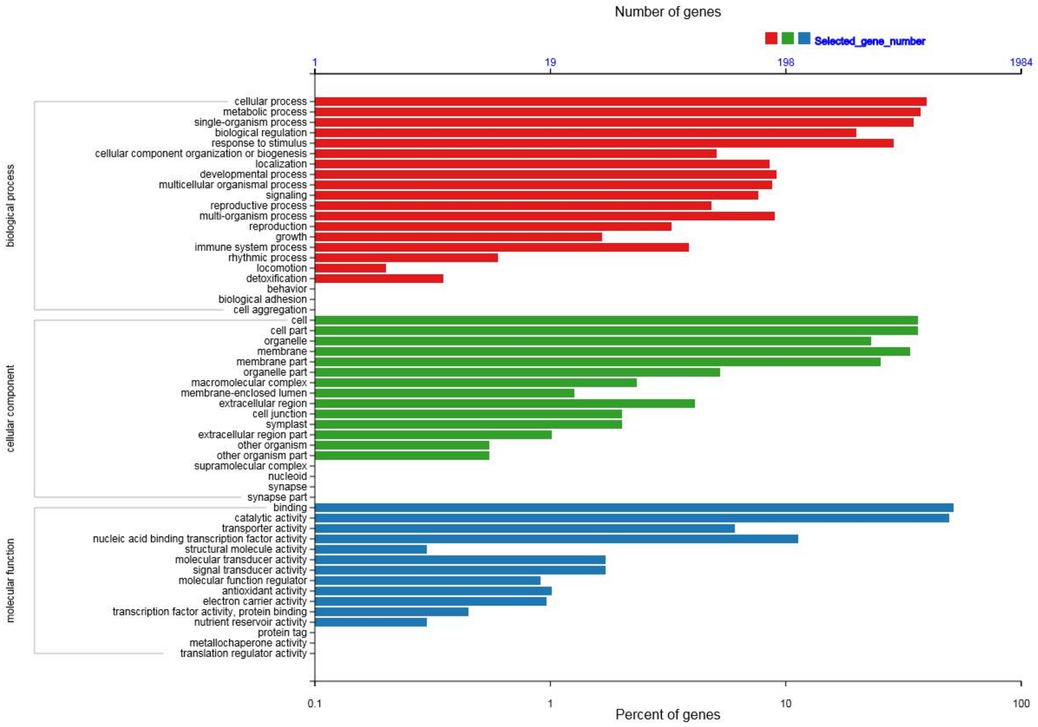

0h_vs_3h

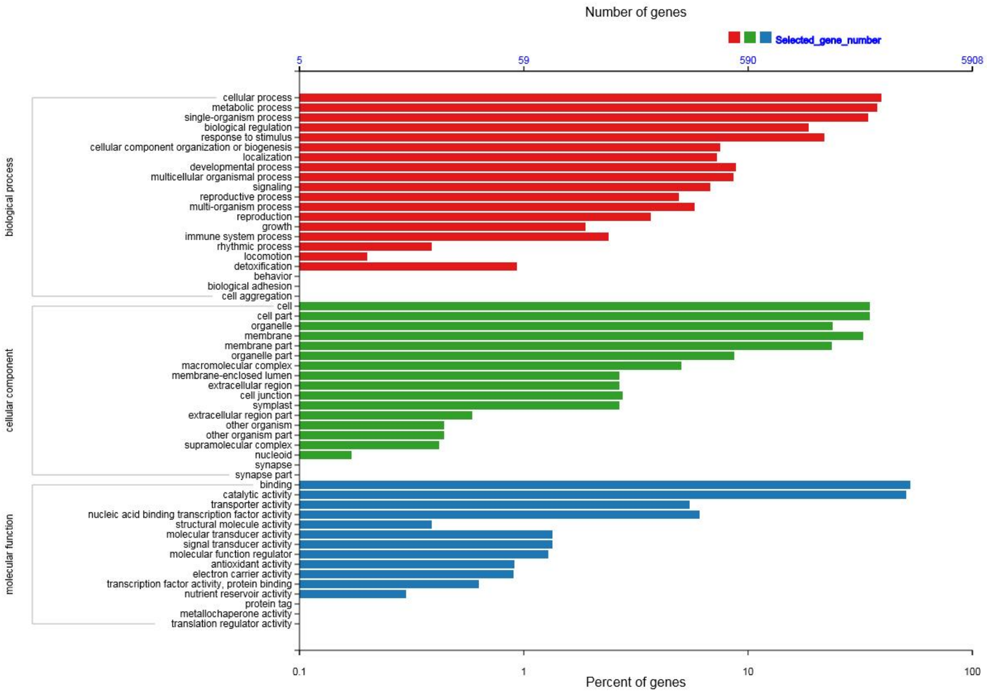

0h_vs_6h

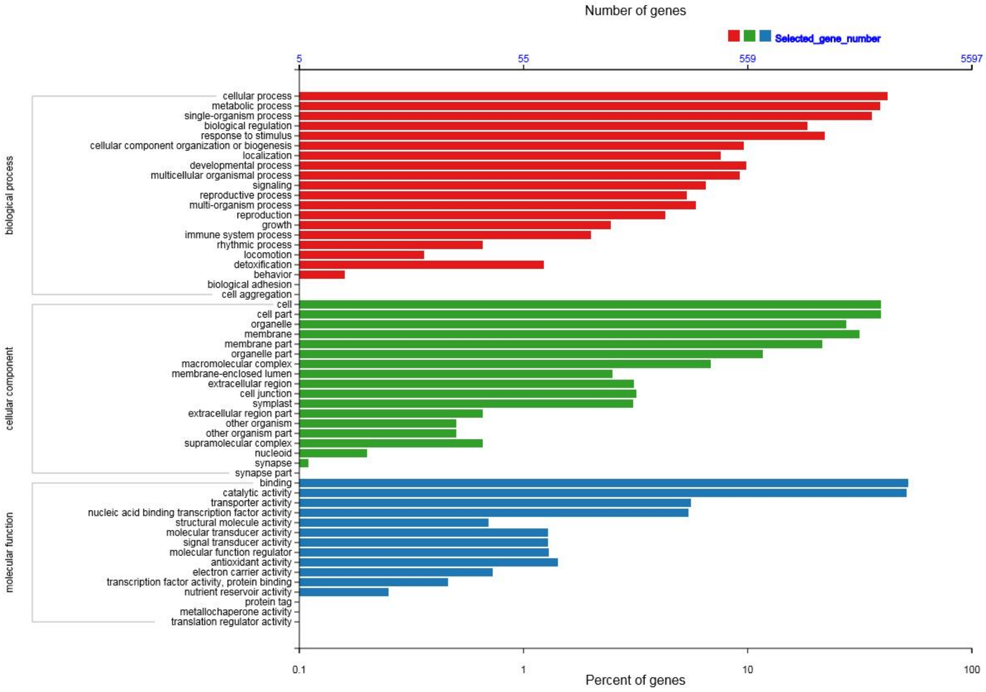

0h_vs_12h

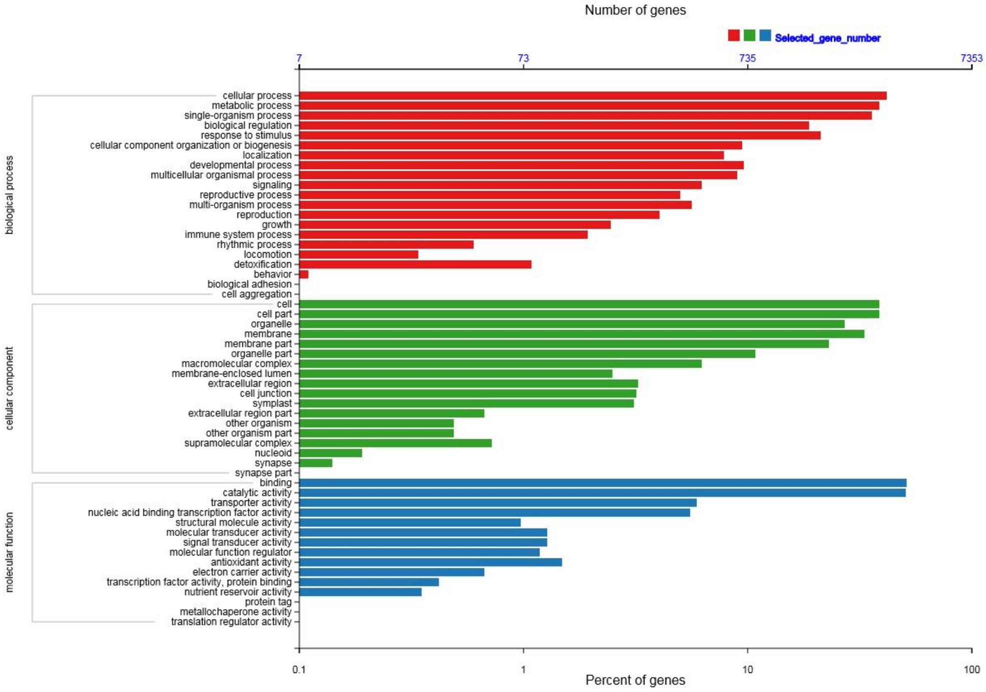

0h_vs_24h

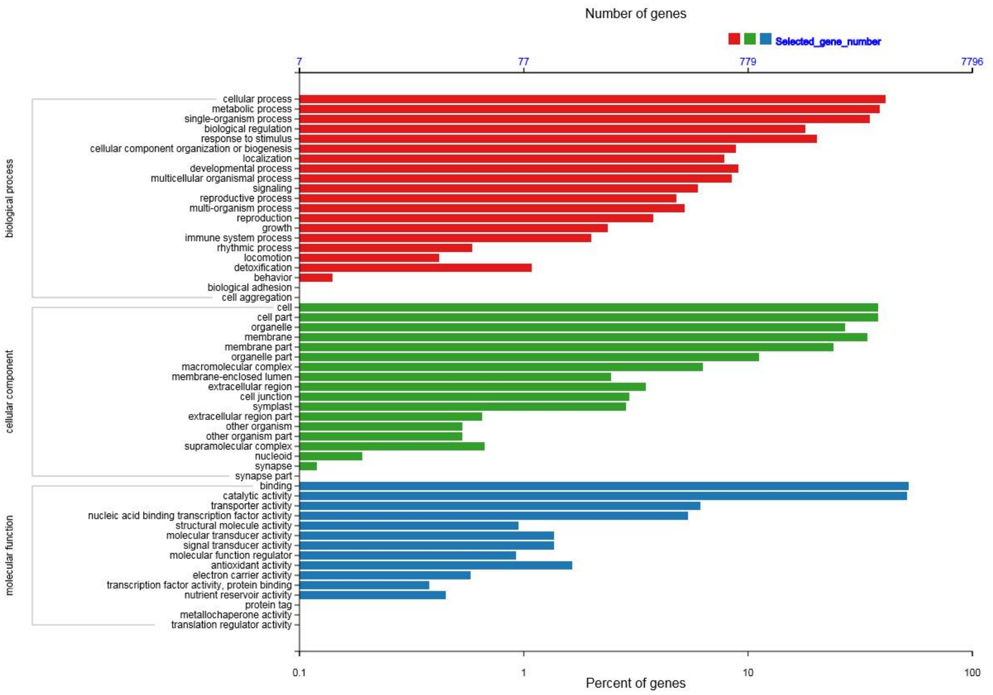

0h_vs_UV (1h, 3h, 6h, 12h, 24h)

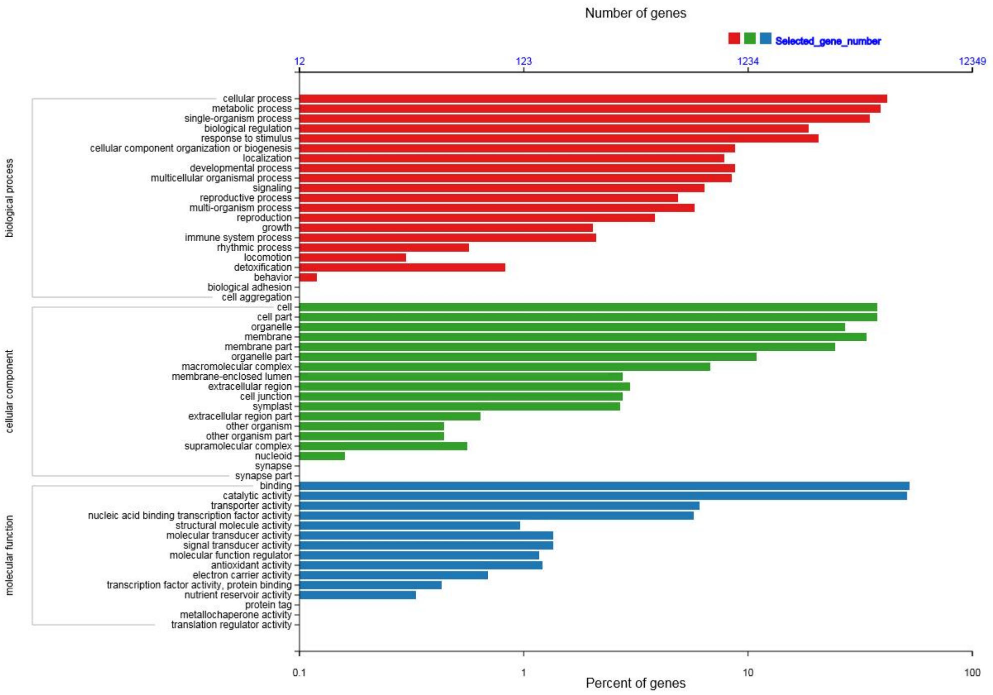

Supplementary Figure S1 | GO classifications of DEGs under UV-B treatment in three main categories: biological process (red), cellular component (green) and molecular function (blue).
