# Supplementary material for: UV-B induces the expression of flavonoid biosynthetic pathways in blueberry (Vaccinium corymbosum) calli
Source: Front Plant Sci. 2022 Nov 22;13:1079087. doi: 10.3389/fpls.2022.1079087 (PMC9722975; doi:10.3389/fpls.2022.1079087)
Supplement: Supplementary file 2 [file DataSheet_2.pdf]

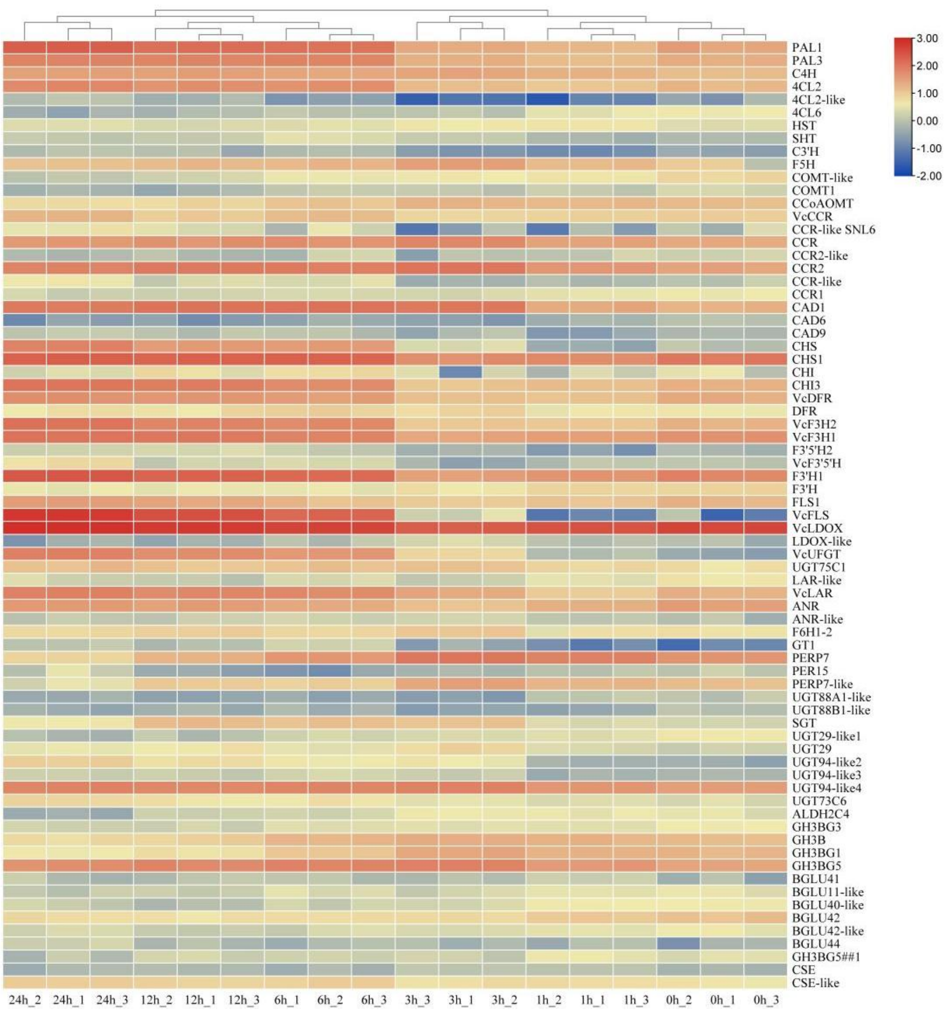

Supplementary Figure S2 | Heatmap analysis for differential expression levels of genes from the phenylpropanoid, flavonoid, flavonol and anthocyanin KEGG pathways. The gradation from red to blue represents the transition from large to small values of $\log_{10}$ (FPKM).
