# Supplementary material for: UV-B induces the expression of flavonoid biosynthetic pathways in blueberry (Vaccinium corymbosum) calli
Source: Front Plant Sci. 2022 Nov 22;13:1079087. doi: 10.3389/fpls.2022.1079087 (PMC9722975; doi:10.3389/fpls.2022.1079087)
Supplement: Supplementary file 3 [file DataSheet_3.pdf]

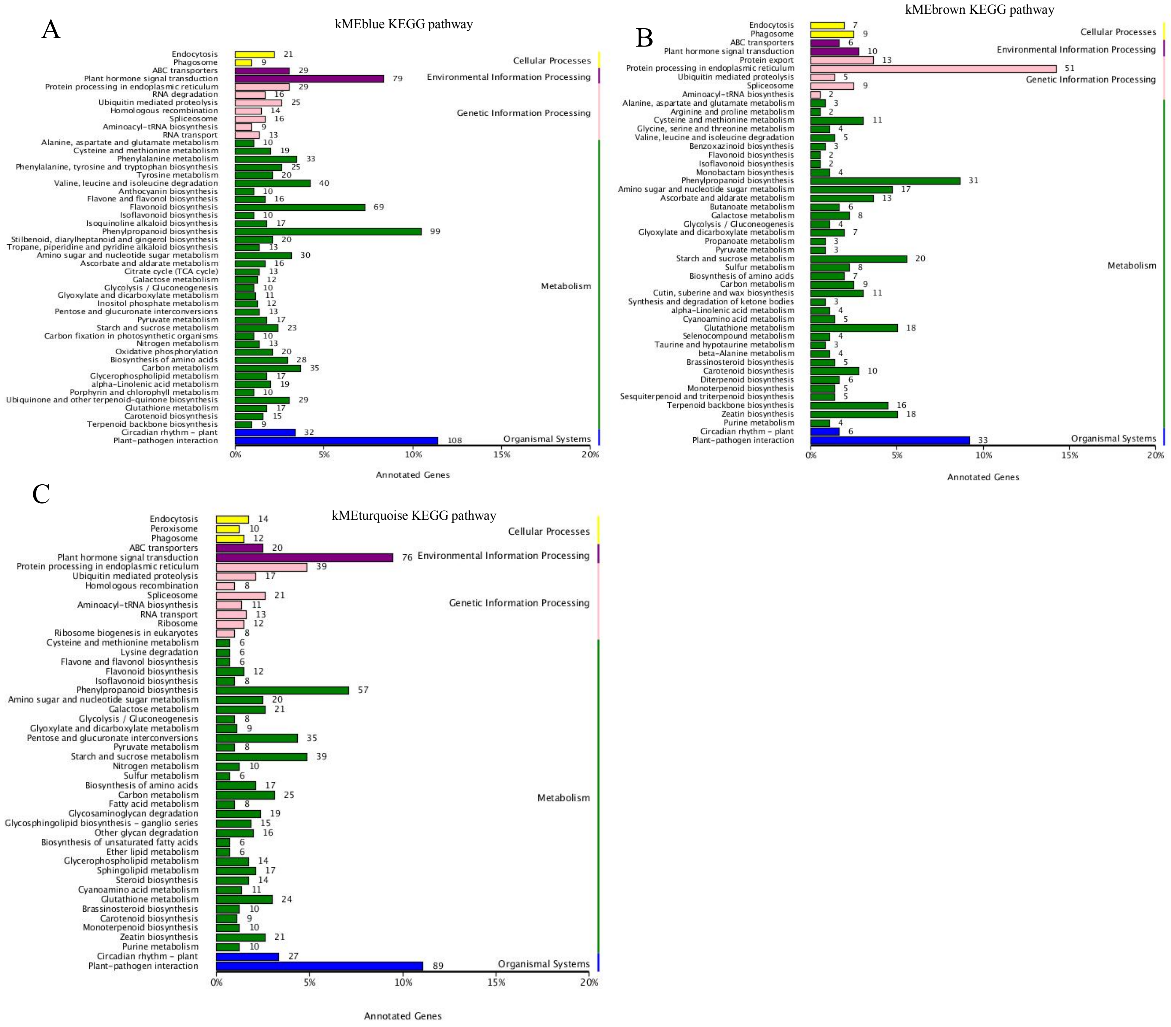

Supplementary Figure S3 | KEGG enrichment pathway analysis for the kMEblue (A), kMEbrown (B) and kMEquoise (C) modules from the weighted gene co-expression network.
