# Supplementary material for: UV-B induces the expression of flavonoid biosynthetic pathways in blueberry (Vaccinium corymbosum) calli
Source: Front Plant Sci. 2022 Nov 22;13:1079087. doi: 10.3389/fpls.2022.1079087 (PMC9722975; doi:10.3389/fpls.2022.1079087)
Supplement: Supplementary file 4 [file DataSheet_4.pdf]

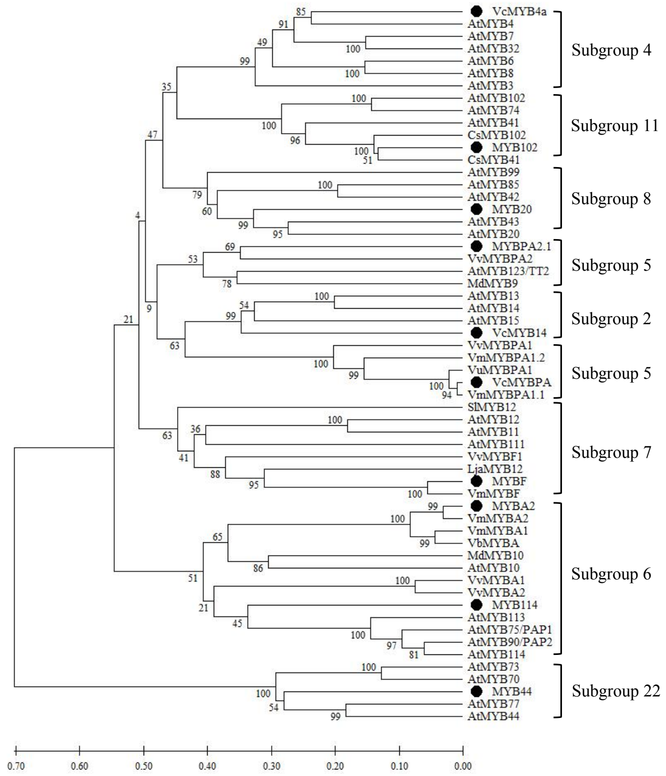

Supplementary Figure S4 | The phylogenetic tree of ten MYB transcription factors identified from the kMEblue module according to weighted gene co-expression network analysis (WGCNA) and other R2R3-MYB subgroup members. Numbers at each interior branch indicate Bootstrap support of 1000 replicates. The scale indicates average number of amino acid substitutions per site. The black dots indicate the MYBs in this study. GenBank accession numbers are as follows: VcMYB4a (AKQ98325), VcMYB14 (URH24710), VcMYBPA (ALP43796), AtMYB4 (AAC83582), AtMYB7 (AAG42001), AtMYB32 (NP_195225), AtMYB8 (NP_849749), AtMYB3 (NP_564176), AtMYB102 (NP_567626), AtMYB74 (NP_192419), AtMYB41 (NP_194540), CsMYB102 (XP_028059323), CsMYB41 (XP_028068496), AtMYB99 (NP_201038), AtMYB85 (NP_567664), AtMYB42 (NP_567390), AtMYB43 (NP_197163), AtMYB20 (NP_176797), VvMYBPA2 (ACK56131), AtMYB123 (NP_188966), MdMYB9 (ABB84757), AtMYB13 (NP_172108), AtMYB14 (NP_188966), AtMYB15 (NP_188966), VvMYBPA1 (CAJ90831), VmMYBPA1.2 (QWW89543), VuMYBPA1 (AKC94840), VmMYBPA1.1 (QWW89542), SlMYB12 (ACB46530), AtMYB12 (ABB03913),

AtMYB11 (NP_191820), AtMYB111 (NP_199744), VvMYBF1 (ACV81697), LjiMYB12 (QER90717), VmMYBF (QWW89550), VmMYBA2 (QWW89534), VmMYBA1 (QWW89533), VbMYBA (UHY14105), MdMYB10 (ACQ45201), AtMYB10 (NP_187888), VvMYBA1 (BAD18977), VvMYBA2 (BAD18978), AtMYB113 (OAP11934), AtMYB75 (AAG42001), AtMYB90 (NP_176813), AtMYB114 (AEE34502), AtMYB73 (NP_195443), AtMYB70 (NP_179910), AtMYB77 (NP_190575), and AtMYB44 (NP_201531).
