# Supplementary material for: UV-B induces the expression of flavonoid biosynthetic pathways in blueberry (Vaccinium corymbosum) calli
Source: Front Plant Sci. 2022 Nov 22;13:1079087. doi: 10.3389/fpls.2022.1079087 (PMC9722975; doi:10.3389/fpls.2022.1079087)
Supplement: Supplementary file 5 [file DataSheet_5.pdf]

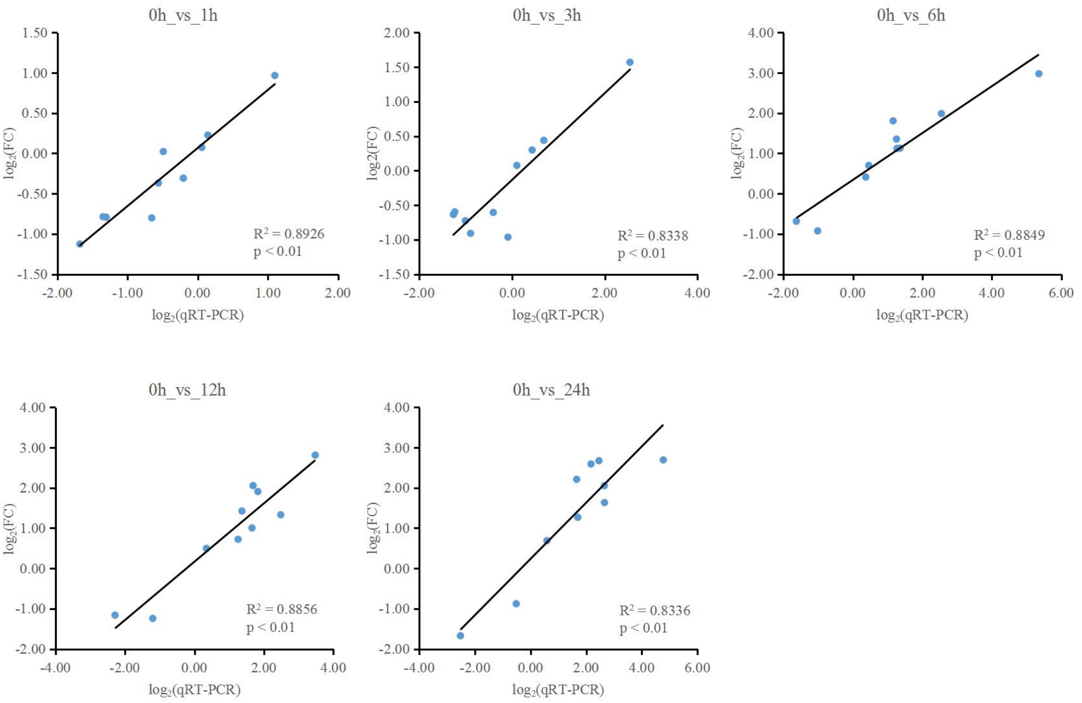

Supplementary Figure S5 | Validation of transcript expression changes by qRT-qPCR. The figure is based on $\log_2(2^{-\Delta\Delta Ct})$ data from qRT-PCR and $\log_2$(fold change) data from RNA-seq. The linear trend line and the R^2 are shown.
